# Biosynthetic Conversion of Ag^+^ to highly Stable Ag^0^ Nanoparticles by Wild Type and Cell Wall Deficient Strains of *Chlamydomonas reinhardtii*

**DOI:** 10.3390/molecules24010098

**Published:** 2018-12-28

**Authors:** Ashiqur Rahman, Shishir Kumar, Adarsh Bafana, Si Amar Dahoumane, Clayton Jeffryes

**Affiliations:** 1 Nanobiomaterials and Bioprocessing Laboratory (NABLAB), Dan F. Smith Department of Chemical Engineering, Lamar University, Beaumont, TX 77710, USA; arahman2@lamar.edu (A.R.); skumar1@lamar.edu (S.K.); abafana@lamar.edu (A.B.); 2 School of Biological Sciences and Engineering, Yachay Tech University, Hacienda San José s/n, San Miguel de Urcuquí 100119, Ecuador; sdahoumane@yachaytech.edu.ec

**Keywords:** *Chlamydomonas reinhardtii*, biosynthesis, AgNPs, colloidal stability, kinetics, conversion, AgNP dissolution, nanofluids

## Abstract

In the current study, two different strains of the green, freshwater microalga *Chlamydomonas reinhardtii* bioreduced Ag^+^ to silver nanoparticles (AgNPs), which have applications in biosensors, biomaterials, and therapeutic and diagnostic tools. The bioreduction takes place in cell cultures of *C. reinhardtii* at ambient temperature and atmospheric pressure, thus eliminating the need for specialized equipment, harmful reducing agents or the generation of toxic byproducts. In addition to the visual changes in the cell culture, the production of AgNPs was confirmed by the characteristic surface plasmon resonance (SPR) band in the range of 415–425 nm using UV-Vis spectrophotometry and further evolution of the SPR peaks were studied by comparing the peak intensity at maximum absorbance over time. X-ray diffraction (XRD) determined that the NPs were Ag^0^. Micrographs from transmission electron microscopy (TEM) revealed that 97 ± 2% AgNPs were <10 nm in diameter. Ag^+^ to AgNP conversion was determined by inductively coupled plasma atomic emission spectroscopy (ICP-AES). The AgNPs were stable over time in the cell culture media, acetone, NaCl and reagent alcohol solutions. This was verified by a negligible change in the features of the SPR band after t > 300 days of storage at 4 °C.

## 1. Introduction

Silver nanoparticles (AgNPs) have numerous applications in medicine, environment, food and agriculture [1,2,3,4]. AgNPs have unique properties to serve as biosensors, catalysts, solvent based nanofluids superconductors, and theranostic tools [3,5,6,7,8]. These AgNPs can be synthesized by chemical [9,10], physical [11] and biological routes [12,13]. The traditional chemical and physical processes require large energy inputs and generate toxic byproducts [14]. In contrast, the biosynthesis of AgNPs using biological materials as the reducing and stabilizing agents is an eco-friendly, low-cost process [15,16] with reduced environmental impacts [17,18].

Various biological resources, including algae [19,20], bacteria [21,22], yeast [23,24], fungi [25,26] and plants [27,28,29], have been used to synthesize various inorganic NPs. Under certain conditions, these resources allow the control of the shape, size and composition of the produced nanomaterials. Among all the various biological platforms, algae and their extracts have recently attracted growing attention for the biosynthesis of nanomaterials [12].

Various species of microalgae have been screened for the synthesis of numerous nanomaterials [30]. For example, cyanobacteria [31], *Charophyta* [16], *Chlorophyta* [32], diatoms [33,34] and *Euglenozoa* [35] have all been used to produce AuNPs. Aside from being a model for genetic studies, *Chlamydomonas reinhardtii*, a green, freshwater microalga, has been previously characterized for its ability to produce nanomaterials [12]. Moreover, various strains of *C. reinhardtii* with different physical and biological characteristics (size, cell wall, motility, etc.) are readily available [36]. For instance, living cultures of this species were successfully used to produce bimetallic alloy NPs made of silver and gold with well controlled compositions [37]. In a different study, both the cell free extract and collected whole cells of *C. reinhardtii* were used to synthesize AgNPs [15].

Theoretical mechanisms have been proposed to explain the biosynthesis of nanoparticles by *C. reinhardtii* and other microalgae [19,20,38]. The synthesis can be intracellular or extracellular. The secretion of reducing agents into the growth medium can induce the formation of metallic NPs from aqueous solutions of the corresponding salts [39]. Barwal et al. found that the intracellular synthesis of AgNPs using harvested whole cells of *C. reinhardtii* was faster than using cell-free extracts [15]. Although different sets of process conditions were used for the two syntheses, the faster intracellular synthesis could be due to the physiological regeneration of reducing equivalents by the photosynthetic apparatus [40]. Furthermore, AgNP synthesis by living cells of *C. reinhardtii* is proportional to the Ag^+^ concentration at low concentrations of 20–500 nM [41]. However, the production rate of AgNPs using nM silver salt precursors is too low to make large-scale production economically feasible, so higher concentrations are required [19]. Therefore, the production, conversion, and stability of AgNPs produced at higher Ag^+^ concentrations must be presented in this work.

In the current study, two strains of *C. reinhardtii*, i.e., the wild type (WT) strain (CC1690) and the cell wall deficient (CWD) strain (CC4425), were used to synthesize AgNPs in flask cultures after the addition of aqueous AgNO_3_ at 2 different final concentrations. Previous studies have shown that NP formation can be intracellular as particles localize within the entire cell including the thylakoids where reducing agents are generated [15,16]. However, the cell wall can be a barrier to intracellular synthesis because the outer cell wall can have an adsorption affinity for metal cations, thus hindering transport into the cytoplasm [42,43]. While the CWD strain of *C. reinhardtii* lacks a cell wall, it exhibits normal photosynthetic behavior [44]. Moreover, it is capable of biosynthesizing cell wall constituents at a rate matching that of the wild type, but cannot assemble them to form cell walls [45]. Bearing in mind this sole difference between the two strains of *C. reinhardtii* in this study, it is necessary to compare the outcomes of the AgNP synthesis process, i.e., the Ag^+^ to the AgNP conversion to completely understand the synthesis process and shed light on the impact of the cell wall on such processes. The current study measured that the presence or absence of the cell wall does not affect AgNP conversion or production rate at 1.250 mM and 0.625 mM precursor (AgNO_3_) concentrations. Additionally, the presence or absence of the cell wall has no impact on the stability of the as-produced AgNPs, monitored by analyzing the evolution of the SPR band in saline solution, acetone and reagent alcohol after more than 10-months of storage in the dark at 4 °C. These highly stable AgNPs can be a potential source for AgNP dissolution studies [46,47]. As reported previously, environmental AgNP dissolution studies have been complicated by a lack of NP stability in saline solutions. The long-term stability of our biologically synthesized AgNPs at elevated saline concentrations could enable such dissolution studies. Additionally, these AgNPs can be used as stable nanofluids for thermal conductivity-based applications [6,48,49,50]. Furthermore, our long-term stable AgNPs may find their potential use as AgNP-based drugs that are currently under clinical trials [51,52]. Nevertheless, the greater significance of these stability tests lies in the future exploitation of these AgNPs.

## 2. Results and Discussion

### 2.1. Biosynthesis of AgNPs by C. reinhardtii

**Figure 1:**
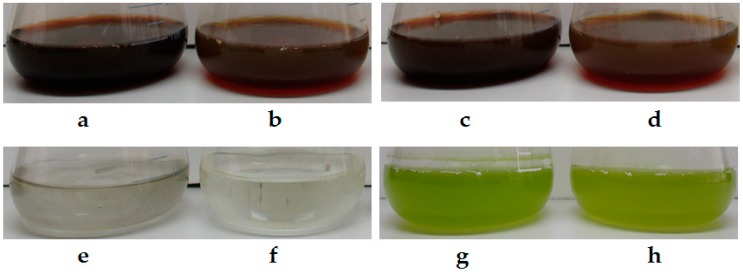
Photographic images of 500 mL Erlenmeyer flasks after 48 h of cultivation. The strain (WT or CWD) and concentration of AgNO_3_ are indicated for each flask. (**a**) WT-1.250 mM; (**b**) WT-0.625 mM; (**c**) CWD-1.250 mM; (**d**) CWD-0.625 mM; (**e**) BBM + 1.250 mM; (**f**) BBM + 0.625 mM; (**g**) WT-0.000 mM; and (**h**) CWD-0.000 mM.

Digital images of the as-synthesized AgNPs by the WT and CWD strains of *C. reinhardtii* after 48 h of cultivation in the presence of cationic silver are presented in Figure 1a–h. The characteristic color change of the cultures from green to dark brown (Figure 1a–d) after the addition of AgNO_3_ indicated the synthesis of AgNPs at both concentrations by both strains [15,53,54]. Cell-free flasks (Figure 1e,f) with the culture media, Bold’s Basal Medium (BBM), and AgNO_3_ showed no NP synthesis but a precipitate of Ag salts. Ag-free cell culture controls (Figure 1g,h) maintained their green color. Similar color of the AgNP colloid was reported via the biosynthesis process using, for instance, bacteria [55,56], fungi [25,57] and plant cell cultures [58,59].

**Figure 2:**
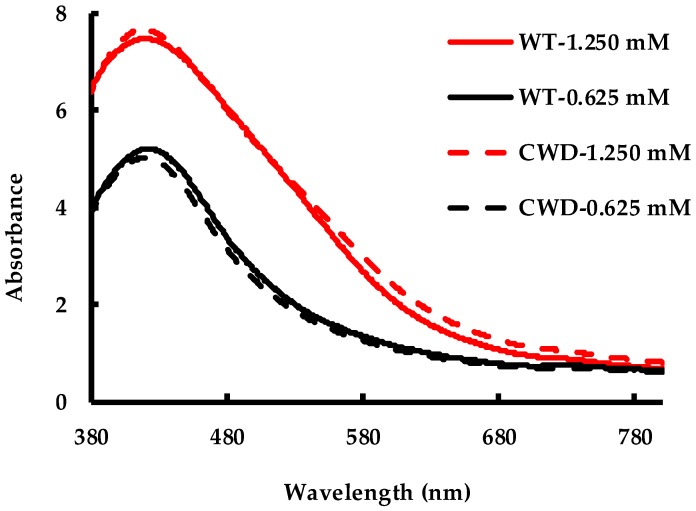
Spectrophotometric measurements of WT and CWD strains at 1.250 mM and 0.625 mM AgNO_3_ concentrations at 192 h.

Color change as a function of AgNO_3_ addition into the cultures (Figure 1a–d) was quantified by UV-Vis spectrophotometry from 380 nm to 800 nm. The surface plasmon resonance (SPR) peak of AgNPs was observed at ~420 nm confirming the presence of AgNPs in the culture medium (Figure 2). The SPR peak for algal-synthesized AgNPs at 420 nm has been reported previously [15,60]. According to Figure 2, each spectrum has a bell shape indicating that the AgNPs were spherical [61]. Additionally, at the higher concentration (1.250 mM), the SPR peak displayed a shoulder at ~490 nm that may be attributed to the strong interaction between the particles [62] or as a result of a heterogeneous AgNP size distribution [63]. In contrast, a more symmetric peak, observed at the lower concentration (0.625 mM), implies less or no significant interaction between the AgNPs. As reported previously [32], the intensity of the SPR band at the higher concentration of AgNO_3_ is greater than at the lower concentration in both cases. The recorded spectra for both strains at a given concentration appear to be similar in shape and intensity. Therefore, the cell wall has no impact on the as-produced AgNPs. No absorbance was recorded at ~420 nm for the control flasks containing no cells (Figure S2 in the Supplementary Materials), which was consistent with their physical appearance (Figure 1e,f).

**Figure 3:**
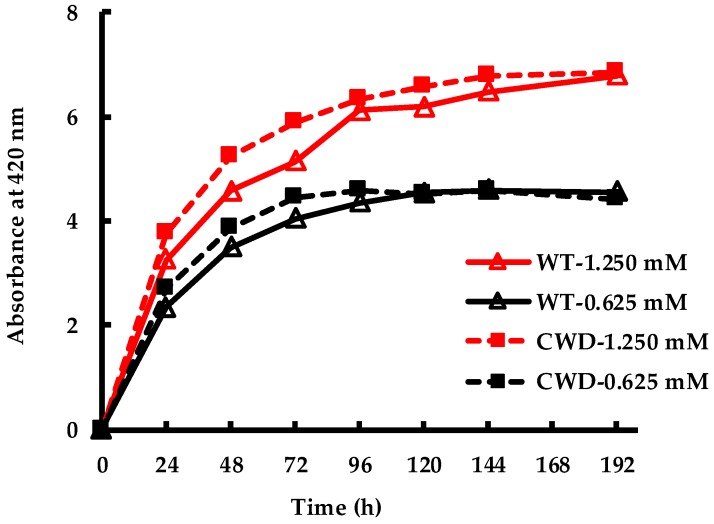
Evolution of AgNP SPR peak vs. time for WT and CWD strains at 1.250 mM and 0.625 mM AgNO_3_ concentrations.

The difference between the spectrophotometric absorbance at 420 nm and 800 nm, a measure of AgNPs present in the culture, is plotted vs. time in Figure 3. For both strains at 0.625 mM, the SPR peak plateaued at an absorbance of ~4.5 and the synthesis was complete by 96 h. In contrast, at 1.250 mM the synthesis took longer for both strains, the absorbance continued to increase until 192 h, and its value asymptotically tended toward a maximum of ~6.8. These results could be explained by (i) the higher ratio of biological material to Ag^+^ at 0.625 mM compared to at 1.250 mM led to the more rapid depletion of the precursor (cf. Table S1 in the Supplementary Materials); (ii) the maximum SPR peak values (~6.8) were 49% (for WT) and 55% (for CWD) higher than the respective peak values (~4.5) at 0.625 mM instead of 100% as the initial AgNO_3_ concentration doubled from 0.625 mM to 1.250 mM.

In the current study, over 50% of total synthesis at both concentrations (i.e., 1.250 mM and 0.625 mM) were completed within the first 24 h under constant illumination of 69 ± 5 μE m^−2^ s^−1^ incident to the flask surface. In a similar study, Barwal et al. used washed biomass of *C. reinhardtii* and reported a completed conversion of 1 mM Ag^+^ to AgNP in 10 h [15]. However, in the same study, cell free extract of *C. reinhardtii* took 13 days in the dark to go to completion. Another study, exposing whole living cultures of *C. reinhardtii* to 0.1 mM AgNO_3_ under a photoperiod of 16 h light/8 h dark, but without providing information of the light intensity, reported the AgNP SPR peak to plateau within 48 h [37]. These findings possibly indicate a combined impact of the initial precursor concentration, available biomass and the extent of illumination on the AgNP synthesis process. More importantly, both *C. reinhardtii* strains in our study showed a similar trend in the SPR peak evolution which may imply that the cell wall of the WT strain does not impact the Ag^+^ to AgNP conversion.

### 2.2. AgNP Morphological and Crystallographic Characterization

**Figure 4:**
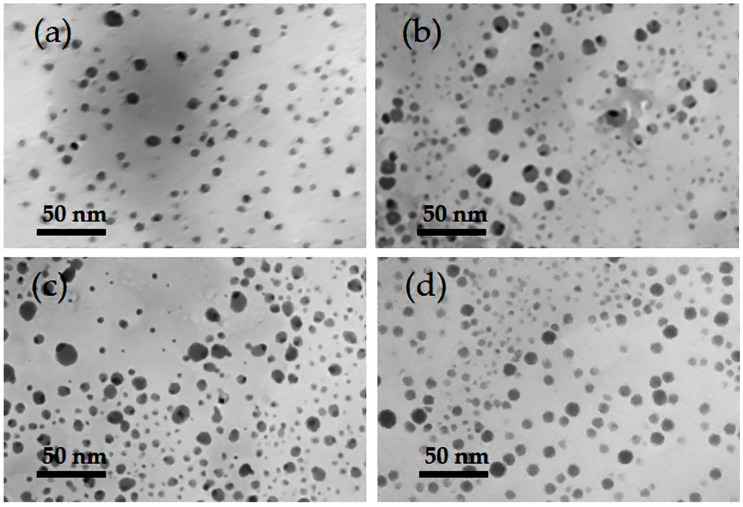
TEM micrographs of AgNPs produced by: (**a**) WT-1.250 mM; (**b**) WT-0.625 mM; (**c**) CWD-1.250 mM; and (**d**) CWD-0.625 mM.

**Figure 5:**
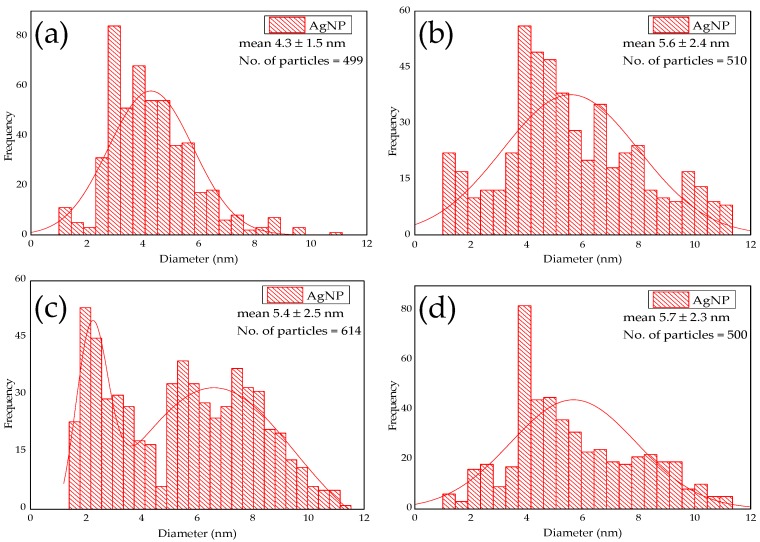
Mean particle size distribution of AgNPs produced by: (**a**) WT-1.250 mM; (**b**) WT-0.625 mM; (**c**) CWD-1.250 mM; and (**d**) CWD-0.625 mM.

The Transmission Electron Microscopy (TEM) revealed that the as-produced AgNPs were well-dispersed and mostly spherical (Figure 4a–d). In addition, Figure 5a–d shows the mean particle size distribution for each of the micrographs. The WT strain at 1.250 mM and 0.625 mM AgNO_3_ concentrations produced AgNPs with a size range of 4.3 ± 1.5 nm (n = 499) and 5.6 ± 2.4 nm (n = 510), respectively, while CWD strains at these concentrations produced AgNPs of 5.4 ± 2.5 nm (n = 614) and 5.7 ± 2.3 nm (n = 500) in size, respectively. This agrees with the size range of AgNPs produced by the *C. reinhardtii,* even if bigger particles of 35 ± 5 nm size were also reported [15]. In the current study, 97 ± 2% of all produced AgNPs by both strains and for both concentrations were <10 nm. To the best of our knowledge, only a few algal strains are able to synthesize AgNPs below this limit [12]. However, the size distributions confirm the presence of a heterogenous population of AgNPs for all the samples. Particularly, the sample CWD-1.250 clearly shows a bimodal distribution (Figure 5c) which can be due to the formation of AgNP aggregates [64,65,66]. This was also noted by the shoulder in the spectrophotometric scans for the samples at 1.250 mM, which had a higher number of AgNPs. Although these two distinct populations have their size centered at less than 10 nm, we think this heterogeneity is due to the concentration dependent toxicity effect of cationic silver that may induce cell lysis by attacking the cell membrane and releasing cell components into culture media hence causing partial loss of cell control on NP biosynthesis [35,37]. However, we think that one possible way to reduce the cytotoxicity can be achieved by a chelation process [67,68]. This can be done by culturing *C. reinhardtii* cells at very low AgNO_3_ concentrations. This will lead to chelation by forming Ag-organic matter molecules that, in turn, will induce leaching of Ag compounds from the cell surface.

**Figure 6:**
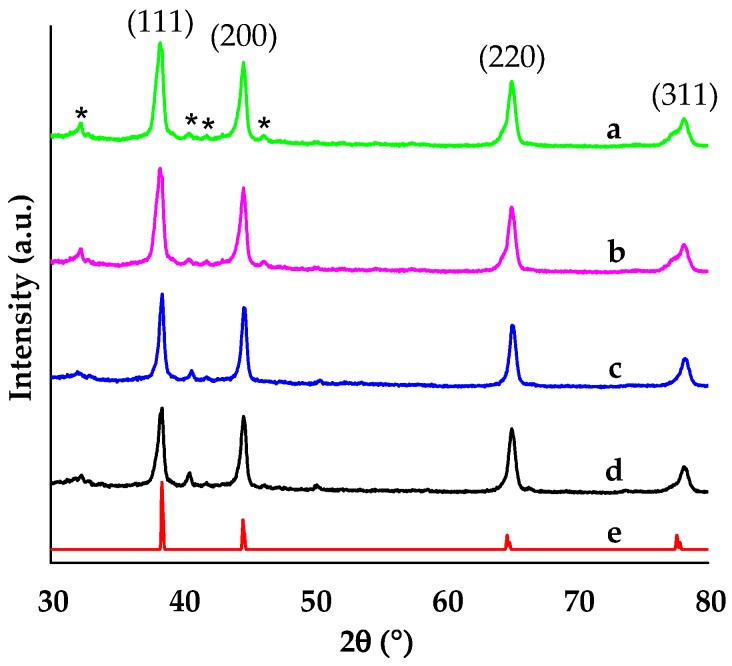
X-ray diffraction pattern of AgNPs produced by: (**a**) WT-1.250 mM; (**b**) CWD-1.250 mM; (**c**) WT-0.625 mM; (**d**) CWD-0.625 mM; and (**e**) Ag metal. * These may be attributed to the salts of the BBM culture media.

Figure 6 shows the XRD pattern for the as-produced AgNPs. The characteristic 2θ peaks at 38.40, 44.66, 65.07 and 78.18°, respectively, correspond to the (111), (200), (220) and (311) planes of the face-centered cubic (fcc) cell lattice of metallic silver (JCPDS files no. 03-0921) as obtained by Manikprabhu and Lingappa [69]. Similar results obtained by Kumar et al. [70] validate the presence of metallic silver that was stabilized by biological molecules. The higher intensity of the peak at higher AgNO_3_ concentrations reiterates the higher AgNP formation at higher initial precursor concentration, as described previously by the greater SPR peak absorbance at higher AgNO_3_ concentration (Figure 2). Four weak peaks, indicated by *, can be seen in all the diffractograms. These may be attributed to the salts of the BBM culture media.

### 2.3. Ag^+^ to AgNP Conversion

**Figure 7:**
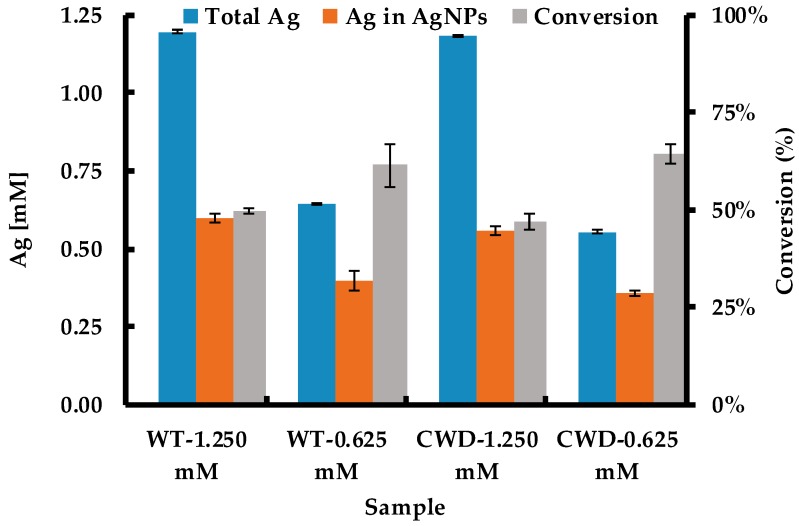
Biosynthetic conversion of Ag^+^ to Ag^0^ (final [Ag^0^] mM/initial [Ag^+^] mM).

Figure 7 shows the initial Ag concentration and the concentration of Ag found in the AgNPs at the completion of synthesis, as measured by ICP-AES. The conversion was then calculated based on these concentrations. The conversion was higher at lower precursor concentrations for both WT and CWD strains. However, in terms of Ag in the AgNPs, the biosynthesis at 1.250 mM produced 51% and 56% more AgNPs than at 0.625 mM for WT and CWD, respectively. These two values agree with the SPR peak intensities at 420 nm (Figure 3). For both species at both concentrations, the conversion for AgNP biosynthesis via the current method does not reach 100%. Several phenomena could explain this outcome: (i) the higher toxicity of Ag^+^, compared to other metallic precursors such as Au^+^, limits the reduction of Ag^+^ to Ag^0^ facilitated by metabolically produced reducing agents [37,40]; (ii) the chloride salts, initially at ~0.56 mM concentration in BBM, partly complex the Ag^+^ to form chloro-complexes (i.e., AgCl, ${\mathrm{AgCl}}_{2}^{-},$ etc.) unavailable for reduction to Ag^0^ [71]; (iii) the complexation of Ag^+^ by the organic matter [72], mainly made of polysaccharides, and by the cell surface; and loss of AgNPs that were bound to sedimentable biological materials. Additionally, the differences between the measured Ag inputs and the theoretical inputs can be due to the adsorption of Ag^+^ or AgNPs to the centrifuge tubes or pipettes, volume loss and carryover during sample transfer for dilution and measurement, as reported previously [70].

### 2.4. Colloidal Stability of Biosynthetic AgNPs

#### 2.4.1. AgNP Stability in NaCl

**Figure 8:**
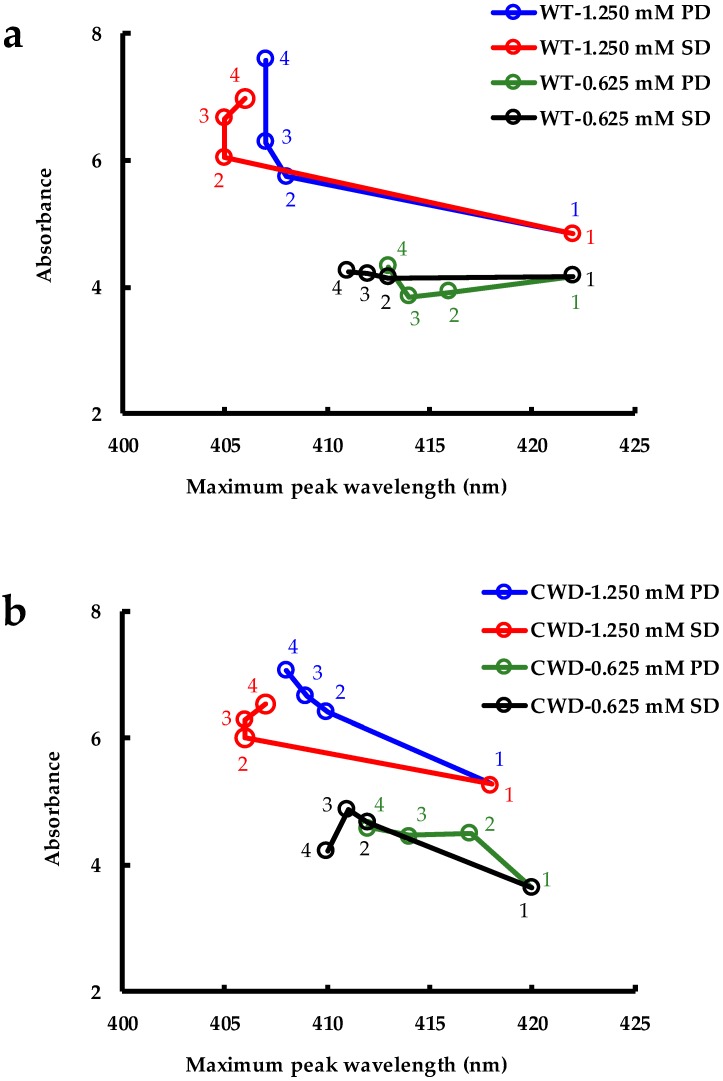
Stability of AgNPs produced by: (**a**) WT strain at 1.250 mM and 0.625 mM AgNO_3_ concentrations after NaCl addition at physiological dose (PD, 9 g L^−1^) and sea-water dose (SD, 27 g L^−1^); (**b**) CWD strain at 1.250 mM and 0.625 mM AgNO_3_ concentrations after NaCl addition at physiological dose (PD, 9 g L^−1^) and sea-water dose (SD, 27 g L^−1^) (1: before adding NaCl (t = 0); 2: after adding NaCl (t = 15 min); 3: after 18 h; and 4: after 72 h).

The AgNPs produced by converting AgNO_3_ to Ag^0^ by living cultures of *C. reinhardtii* were challenged by aqueous solutions of NaCl at both physiological (9 g L^−1^) and sea-water concentrations (27 g L^−1^) and the SPR band was recorded for each sample. In Figure 8a,b, the difference between the absorbance at the peak of the SPR band (λ_max_), and the absorbance at 800 nm (λ_800_) is plotted vs. λ_max_ at different times after exposing the AgNPs to NaCl.

In all cases, a significant blueshift of the SPR peak occurred with increasing time. For both strains and for the NaCl physiological dose (PD) and sea-water dose (SD), the intensity of the SPR peak of the AgNPs increased significantly for 1.250 mM while it remained constant for WT and slightly increased for CWD at 0.625 mM. The most likely explanation to the blueshift and the increase in the peak intensity (i.e., the AgNP stability) can be given from two points of view: (i) an increase in the electron density of the particles owing to the addition of NaCl as previously reported when AgNPs were exposed to KCl solution [73]. It is well established that an increase in the density of the free electrons in the AgNPs leads to a blueshift of the band [74,75,76]; (ii) the presence of Na^+^ and Cl^−^ that increases electrostatic repulsion between NPs and decreases their aggregation [77]. Guo et al. also observed an increase in the SPR peak absorbance, in all cases, when their AuNPs were exposed to various concentrations of NaCl ranging from 0.43 M to 2.58 M. In the current study, we subjected our AgNPs to lower concentrations of 0.15 M NaCl (for PD) and 0.46 M NaCl (for SD).

As depicted in Figure 8a,b, the overall increase in the absorbance along with the peak wavelength shift could be attributed to the complete or partial removal of the near-field electromagnetic coupling interactions between the dipolar oscillation of the electrons [78]. The disappearance of the shoulder (for 1.250 mM) and the narrowing of the SPR band (Figure S3a,b in the Supplementary Materials vs. Figure 2) further corroborate these facts. These results may potentially find their use in the study of NP dissolution. Previous studies in the field of AgNP dissolution in saline environments have been complicated by aggregation, which can be counteracted by using salt-stable NPs such as those developed in this work [46,47].

#### 2.4.2. AgNP Stability in Acetone

**Figure 9:**
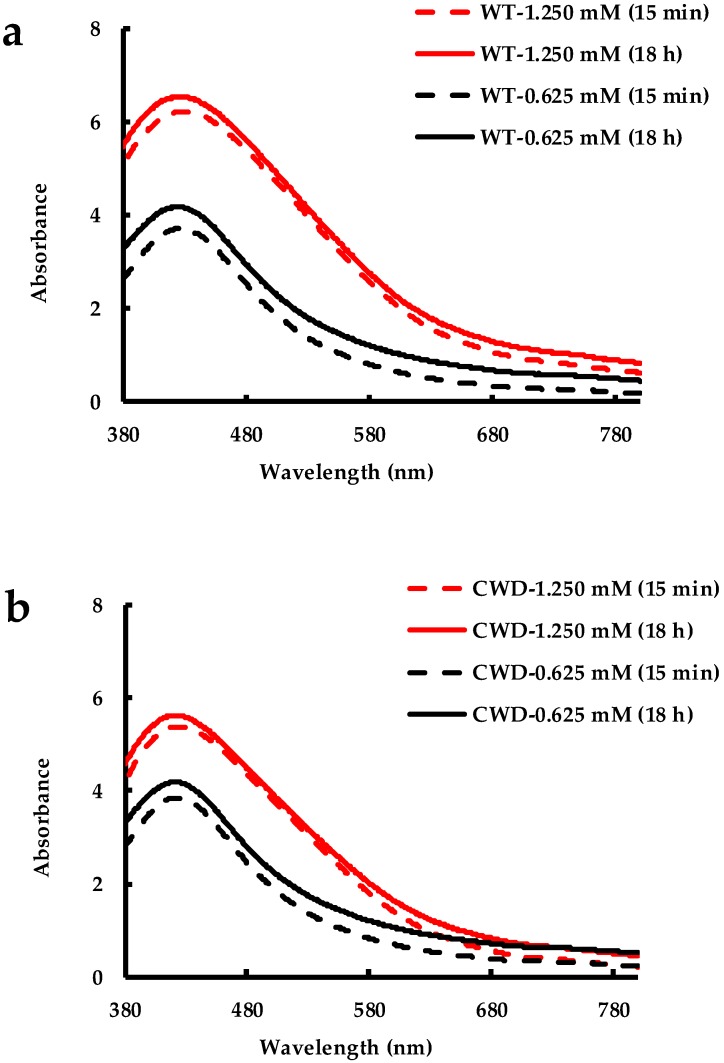
Stability in acetone of AgNPs produced by: (**a**) WT strain at 1.250 mM and 0.625 mM AgNO_3_ concentrations; (**b**) CWD strain at 1.250 mM and 0.625 mM AgNO_3_ concentrations.

Acetone is normally used to separate colloidally stable AgNPs from their mother solution. Sahoo et al. separated their polyvinyl pyrrolidone (PVP) and gelatin stabilized AgNPs by using acetone [79]. They used an equal or higher amount of acetone with their sample to agglomerate and precipitate AgNPs. In our study, all the samples, made of AgNPs synthesized by both *C. reinhardtii* strains, remained stable in 89% acetone. Just after the addition of acetone at 15 min and 18 h later, the SPR peak for both WT and CWD strains exposed to both AgNO_3_ concentrations looked alike and displayed similar shape and intensity (Figure 9a,b). In other words, 18 h were not enough for acetone to dissolve the organic matter surrounding the AgNPs and alter the stability of the latter. This confirms again the high stability of the bioproduced AgNPs by both strains of *C. reinhardtii* at both AgNO_3_ final concentrations. We believe that these AgNPs have the potential to work as acetone-based nanofluid in heat transfer applications and hence they need to be further studied [80,81].

#### 2.4.3. AgNP Stability in Reagent Alcohol

**Figure 10:**
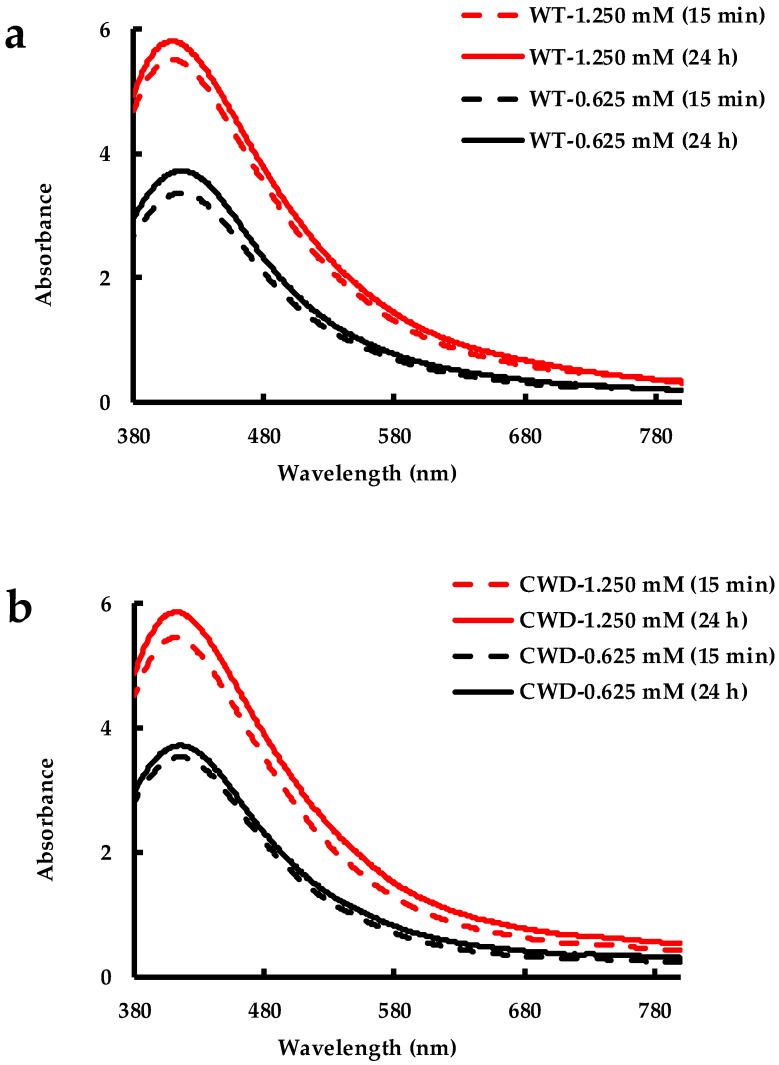
Stability in reagent alcohol of AgNPs produced by: (**a**) WT strain at 1.250 mM and 0.625 mM AgNO_3_ concentrations; and (**b**) CWD strain at 1.250 mM and 0.625 mM AgNO_3_ concentrations.

The solvent reagent alcohol (63–64% ethanol, 3.2–3.9% propanol and 2.9–3.5% methanol) can be used to precipitate colloidally stable NPs from their mother solution for further analyses, such as XRD and TGA [82]. In the current study, the AgNPs produced by both *C. reinhardtii* strains proved to be stable in reagent alcohol even after being subjected to centrifugation at 7500× *g* for 10 min and left to react for another 24 h. The SPR bands for both WT and CWD strains exposed to both AgNO_3_ concentrations, taken at both 15 min and one day after the alcohol addition, looked alike and showed similar shape and intensity (Figure 10a,b). These findings, too, corroborate the high stability of the as-bioproduced AgNPs that can find their use as ethanol-based silver nanofluids [6].

#### 2.4.4. Long-Term AgNP Stability

**Figure 11:**
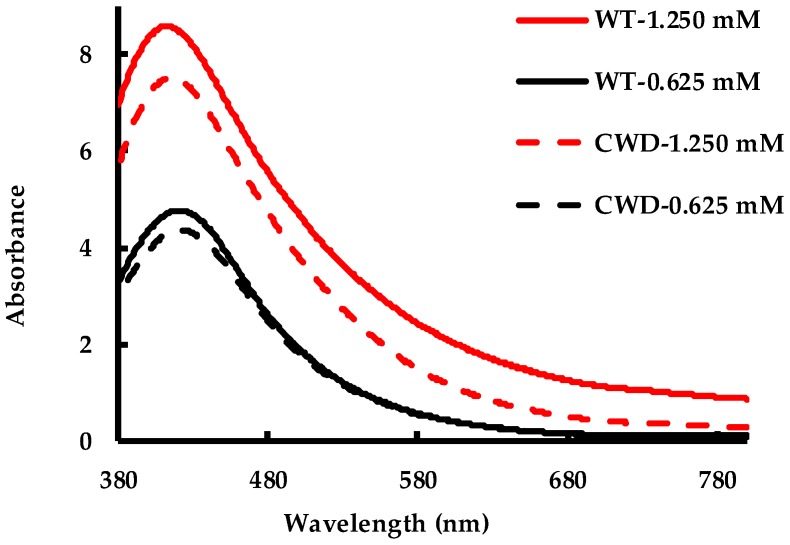
Stability of AgNPs after 10-month storage in the refrigerator.

The recorded SPR bands of the as-produced AgNPs, stored at 4 °C for 10 months, are displayed in Figure 11. Overall, the spectra of the 4 samples highlight their stability. If compared to the spectra recorded 10 months earlier of the freshly synthesized AgNPs (Figure 2), the features of the peaks remained the same for WT-0.625 mM, CWD-1.250 mM and CWD-0.625 mM while they slightly evolved for WT-1.250 mM. For the latter, the SPR peak intensity increased by 10%, the shoulder (at ~490 nm) disappeared and a blueshift of 8 nm was noticed. The blueshift, disappearance of the shoulder and increased peak intensity altogether indicate less interaction between the AgNPs and can be explained by a decrease in the particle size with time [83,84,85,86]. Importantly, the peak intensities for all four samples demonstrate the long-term stability of the as-produced, unprocessed AgNPs. These AgNPs can be used in the fields where long-term storage is necessary. It is well-known that the stability of drug nanoparticles is an important factor when long time storage is concerned [87]. With the current results, we therefore believe that our AgNPs have the potential to be deployed as drug delivery agents [51,52].

Colloidal stability refers to the ability of the inorganic nanoparticles to form stable and homogenous suspensions within their solvents [12]. In the current study, we explored AgNP stability in their mother solvent for t > 300 days as well as in other solvents. We demonstrated that the as-produced AgNPs were stable after 300 days with no decrease in the SPR band intensity and ≤2 nm shift in the wavelength for three out of the four colloidal AgNP samples. This indicates similarity, in terms of colloidal stability, with starch-stabilized AgNPs produced via microwave technology [70], inferring that the biopolymers present in the culture media by both *C. reinhardtii* strains [88,89] could be responsible for long-term colloidal stability. In reference to the previous studies, we think that the possible reducing and stabilizing agents may include various carbohydrate and amine groups (i.e., polysaccharides and proteins) [90,91,92]. Additionally, these outcomes corroborate that the AgNPs synthesized by *C. reinhardtii* remain stable at 4 °C as was previously reported [37]. Moreover, the colloidal stability of AuNPs, produced by *Kirchneriella lunaris*, withstood the preparation of the samples for chlorophyll *a* extraction using the same solvent [38]. Once again, the cell wall had no impact on the stability, synthesis rate or conversion; in both cases, the as-produced AgNPs remain colloidally very stable and withstand the action of salts, solvents and time.

## 3. Materials and Methods

### 3.1. Cell Culture Maintenance and Monitoring

#### 3.1.1. Media Preparation

*C. reinhardtii* were cultured in Bold’s Basal Medium (BBM) which was prepared with several modifications of 3N-BBM+V [93]. The composition of the modified 3N-BBM+V medium was: 430 μmol L^−1^ K_2_HPO_4_, 1.3 mmol L^−1^ KH_2_PO_4_, 300 μmol L^−1^ MgSO_4_·7H_2_O, 2.94 mmol L^−1^ NaNO_3_, 128 μmol L^−1^ CaCl_2_·2H_2_O, 430 μmol L^−1^ NaCl, 132 μmol L^−1^ EDTA, 18 μmol L^−1^ FeSO_4_·7H_2_O, 185 μmol L^−1^ H_3_BO_3_, 4.91 μmol L^−1^ ZnCl_2_, 1.17 μmol L^−1^ MnCl_2_·4H_2_O, 1.01 μmol L^−1^ CuSO_4_·5H_2_O, 280 nmol L^−1^ CoCl_2_·6H_2_O and 794 nmol L^−1^ Na_2_MoO_4_. All chemicals were of analytical grade and purchased from Sigma-Aldrich (St. Louis, MO, USA) or VWR (Radnor, PA, USA); deionized water (DIW) was the solvent. The average pH of the prepared BBM was 6.7 ± 0.2. The BBM was freshly prepared and sterilized by autoclaving at 121 °C and 1 atm gauge for 20 min. The BBM was allowed to cool for 24 h before being used for sub-culturing.

#### 3.1.2. Sub-Culturing of *C. reinhardtii*

*C. reinhardtii* strains, both wild type (WT) and cell wall deficient (CWD), were purchased from the *Chlamydomonas* Resource Center, University of Minnesota, St. Paul, MN, USA. Axenic sub-culturing was done every week in a Labconco purifier clean bench. All materials and BBM were autoclaved prior to use. New generations were prepared by adding 30 mL of previous generation culture to 120 mL of BBM in a 500 mL borosilicate Erlenmeyer flask. The flasks were kept under an average illumination of 69 ± 5 μE m^−2^ s^−1^ provided by cool white LED tubes. The photoperiod was maintained on a 16/8 h light/dark cycle. The ambient temperature was maintained at 22 ± 1 °C.

### 3.2. Ag^+^ to AgNP Bioreduction Process

The cultures were grown for 21 days before carrying out the experiments. The average cell densities were 1.90 × 10^6^ cells mL^−1^ and 1.84 × 10^6^ cells mL^−1^ for WT and CWD, respectively. Additional characteristic cell culture parameters are presented in the Supplementary Materials (Table S1). The experiment started by mixing together two parent flasks, each containing 150 mL culture of the same strain, and then transferring 90 mL culture to each experimental flask. Then, 10 mL stock AgNO_3_ solutions were added to the flasks to reach final AgNO_3_ concentrations of 1.250 mmol L^−1^ and 0.625 mmol L^−1^. As control experiments, two flasks contained only BBM with the two different concentrations of AgNO_3_ and two additional flasks contained only 90 mL of WT and CWD cultures plus 10 mL deionized water without any addition of AgNO_3_. All flasks were separated by 5 cm during cultivation to eliminate any shading of one flask onto another. The average light intensity was 69 ± 5 μE m^−2^ s^−1^. Sampling was done aseptically in the biosafety cabinet. Samples were collected using a 1 mL micropipette or sterile 30 mL disposable syringe, depending on sample volume.

### 3.3. Characterization Techniques

#### 3.3.1. Spectrophotometric Characterization

The spectrophotometric characterization was performed using a Cary-100 Bio UV-Vis spectrophotometer (Agilent Technologies, Santa Clara, CA, USA). Deionized water was the blank for aqueous samples and 89.6% acetone was the blank only when samples contained acetone. All spectrophotometric analyses were carried out in polystyrene cuvettes except for samples containing acetone, which were in quartz. The samples were scanned from 380 nm to 800 nm in 1.00 cm path length cuvettes. The crude reaction samples saturated the spectrophotometer (cf. Figure S1 in the Supplementary Materials), so these samples were diluted 10× with deionized water, their UV-Vis spectra recorded, multiplied by 10 and reported in the current study. The recorded spectra for AgNPs in salt solutions and solvents were multiplied with respective dilution factors and presented. To study the SPR peak evolution, the difference between the maximum peak absorbance at a particular wavelength (λ_max_), and the absorbance at 800 nm (λ_800_) was accounted and plotted vs. time.

#### 3.3.2. Morphological and Crystallographic Analyses

Transmission Electron Microscopy (TEM, JEOL Ltd., Tokyo, Japan) and X-Ray Diffraction (XRD, Thermo-Scientific, Waltham, MA, USA) were used to characterize AgNP morphology. The reaction media was first filtered using a glass microfiber filter (diameter 25 mm, pore size 1.2 µm). TEM samples were prepared by casting 30 μL of filtrate onto the surface of a PELCO^®^ (Fresno, CA, USA) TEM Grid Carbon Type- B (Ted Pella Inc., 3.05 mm O.D., 400 mesh, 0.4 × 2 mm single slot Cu) and air dried for 24 h. The TEM analysis used a JOEL JEM-1400 Plus Transmission Electron Microscope (120 kV, 1 kV step, 69 μA beam current, 100 kX magnification, spot size 1) equipped with embedded Scanning Transmission Electron Microscopy (STEM, JEOL Ltd., Tokyo, Japan). Additionally, the mean particle size distribution from the micrographs were determined using ImageJ software (version 1.8.0,) developed by the National Institutes of Health (NIH), Bethesda, MD, USA.

For XRD, 10 mL of filtrate was lyophilized in a Labconco Freezone freeze dry system for 24 h. The entire amount of the dried sample was analyzed by a Thermo-Scientific ARL Equinox 100 X-ray diffractometer (Waltham, MA, USA). The X-rays were generated at 41 kV and 0.9 mA power on a full 2θ range of 0–117° for 20 min using a Cu Kα radiation source (λ = 1.54 Å).

#### 3.3.3. Conversion Calculation by ICP

Inductively coupled plasma atomic emission spectroscopy (ICP-AES, Shimadzu ICPE-9820, CCD detector, Kyoto, Japan) was used to quantify the conversion of Ag^+^ to AgNP. A 5% HNO_3_ in distilled water solution was used as the blank and the detection of Ag took place at a wavelength of 328.068 nm. A sample of well-mixed bulk from the reaction flask was diluted 20× with the 5% nitric acid solution and analyzed first to assess the total amount of silver (AgNPs plus unreacted Ag^+^). NaCl was added to a second sample to precipitate the unreacted Ag^+^ as AgCl. NaCl was added at the same concentration as the AgNO_3_ added to each cultivation to ensure complete precipitation of unreacted Ag^+^. After 12 h, the NaCl-added sample was centrifuged at 1000× g for 10 min and the supernatant was diluted 20× with the 5% HNO_3_ solution and analyzed by ICP-AES. Ag in the form of AgNPs was not precipitated by the NaCl, as determined by the NaCl stability test (Figure 8) and was not removed by centrifugation. Therefore, the amount of silver measured in the supernatant represented the amount of silver in the bioreduced AgNPs. Each sample was scanned four times and the limit of detection for Ag was 0.3 µg L^−1^.

### 3.4. Stability Tests for the Bioreduced AgNPs

The biosynthesized AgNPs were tested for colloidal stability after exposure to NaCl, acetone and reagent alcohol. For each test, the reaction media was first filtered using the same glass microfiber filter used for morphological analysis and the SPR bands of the AgNPs in the filtrates were measured. Additionally, spectrophotometry was done on the 10-month old (t > 300 days) AgNP samples to check the colloidal stability after long-time storage.

#### 3.4.1. AgNP Stability in NaCl

The colloidal stability of the AgNPs in the filtrate were verified in solutions with NaCl concentrations of 9 g L^−1^ (physiological dose or PD) and 27 g L^−1^ (sea-water dose or SD). All samples were vortexed for 30 s after the addition of NaCl. Spectrophotometric measurements were taken before adding NaCl (t = 0); after adding NaCl (t = 15 min); after 18 h and 72 h to check any change in SPR band due to the addition of salt.

#### 3.4.2. AgNP Stability in Acetone

9 mL of 99.5% acetone were added to 1 mL filtrate. The mixture was vortexed for 30 s and a spectrophotometric measurement was taken 15 min later. The mixture was then left for 18 h and a second measurement was taken to check any evolution of the SPR band.

#### 3.4.3. AgNP Stability in Reagent Alcohol

5 mL of 70% (*v*/*v*) reagent alcohol (VWR analytical grade solvent consisted of 63–64% ethanol, 3.2–3.9% propanol and 2.9–3.5% methanol) were added to an equal volume of filtrate. The mixture was then vortexed for 30 s followed by centrifugation at 7500× *g* for 10 min. Following the centrifugation, a spectrophotometric measurement taken 15 min later. The mixture was then left for 24 h and a second measurement was taken to check for any evolution of the SPR band.

#### 3.4.4. Long-Term AgNP Stability

AgNPs, contained in the reaction mixture where they were synthesized, were stored in the dark at 4 °C for over 10-months and then the reaction mixtures were taken out and vortexed for 15 s. Aliquots were collected and placed in a spectrophotometric cuvette and shelf-stored. After 24 h, spectrophotometric readings of the samples were taken at wavelengths ranging from 380 nm to 800 nm. The AgNP SPR excitation band at ~420 nm was compared to that of the recorded band at 192 h during the experiment.

### 3.5. Statistical Techniques

Four replicates were taken for each of the four ICP-AES samples to calculate the average total Ag, average Ag in AgNPs and the average conversion. The standard deviations were then calculated using the Microsoft Excel software program (version 16.0, Redmond, WA, USA). Finally, the average data were presented with respective error bars. For particle size distribution analyses, frequency histograms were plotted from the raw particle size data obtained by ImageJ software (version 1.8.0, Bethesda, MD, USA). The following equation was used to calculate the bin width for the histograms, where N = square of the number of data values:

(1) $$\mathrm{Bin}\;\mathrm{width}=\frac{\mathrm{maximum}\;\mathrm{value}-\mathrm{minimum}\;\mathrm{value}}{\mathrm{number}\;\mathrm{of}\;\mathrm{bins}\;(N)}$$

Further, the histograms were curve-fitted using Gaussian peak function in OriginPro software (version 9.0, Northampton, MA, USA). The multiple peak fit function was used in the case of fitting bimodal distribution.

## 4. Conclusions and Future Perspectives

In this study, we described the biosynthesis of AgNPs following the addition of aqueous solutions of AgNO_3_ to whole living cultures of WT and CWD of *C. reinhardtii*. Round-shaped particles of less than 10 nm in diameter that crystallize in a fcc cell lattice were produced. AgNP formation, measured by SPR band intensity, is faster at 0.650 mM than at 1.250 mM. However, Ag^+^ to AgNP conversion depends on the concentration, while a well-structured cell wall does not. The maximum conversion of 64%, achieved by CWD strain at 0.625 mM, completed in 96 h. All the as-formed colloids are very stable over time and withstand the action of reagent alcohol (63–64% ethanol, 3.2–3.9% propanol and 2.9–3.5% methanol), 89% acetone, 9 g L^−1^ and 27 g L^−1^ NaCl. This knowledge should contribute to the design of high throughput, fully automated photobioreactors for the biosynthesis of valuable nanomaterials using microalgae as green nanofactories. While reaction parameters should be tuned to enhance Ag^+^ to AgNP conversion, it is important to fully understand the biochemical mechanism that governs the production of AgNPs using living cultures of *C. reinhardtii*, identify the biomolecules that provide the outstanding colloidal stability for the as-produced AgNPs and the nature of the interaction between these inorganic nano-objects and their stabilizing agents, which could enable the large-scale development of various solvent-based nanofluids.

## Supplementary Materials

The following are available online. Table S1: The characteristic cell culture parameters, Figure S1: Saturated spectrophotometer reading by crude samples of WT-1.250 mM and WT-0.625 mM; Figure S2: Spectrophotometric measurements of BBM + 1.250 mM and BBM + 0.625 mM, Figure S3: Spectrophotometric measurements of AgNPs.
