# Supplementary material for: Biosynthetic Conversion of Ag+ to highly Stable Ag0 Nanoparticles by Wild Type and Cell Wall Deficient Strains of Chlamydomonas reinhardtii
Source: Molecules. 2018 Dec 28;24(1):98. doi: 10.3390/molecules24010098 (PMC6337529; doi:10.3390/molecules24010098)
Supplement: Supplementary file 1 [file molecules-24-00098-s001.pdf]

Supplementary Materials: The following are available online at www.mdpi.com/xxx/s1

Cell density, extracted chlorophyll, absorbance and quantum efficiency were measured for both the WT and CWD strains to maintain consistency in the experiment. The cell density was measured using a hemocytometer (0.0025 mm^2, 0.1000 mm) and a Nikon Labophot-2 light-microscope. The maximum quantum efficiency of photosystem II (Φ_{PSII}), an indication of cell culture health and photonic to chemical energy conversion, was determined by PAM fluorometry using a Z985 Cuvette Aquapen Fluorometer (Qubit Biology Inc., Kingston, Ontario, Canada), when the Kautsky Induction (OJIP) curves were recorded for 5 s according to the manufacturer's protocol. The samples were dark adapted for 10 minutes before each measurement.

Table S1. The characteristic cell culture parameters.

Parameters	Wild Type (WT)	Cell Wall Deficient (CWD)
age of culture (days)	21	21
average cell density (cells mL^{-1})	1.90·10^6	1.84·10^6
absorbance of chlorophyll *a* at 663 nm	0.516	0.466
quantum efficiency Φ_{PSII} (at t=0)	0.558	0.552

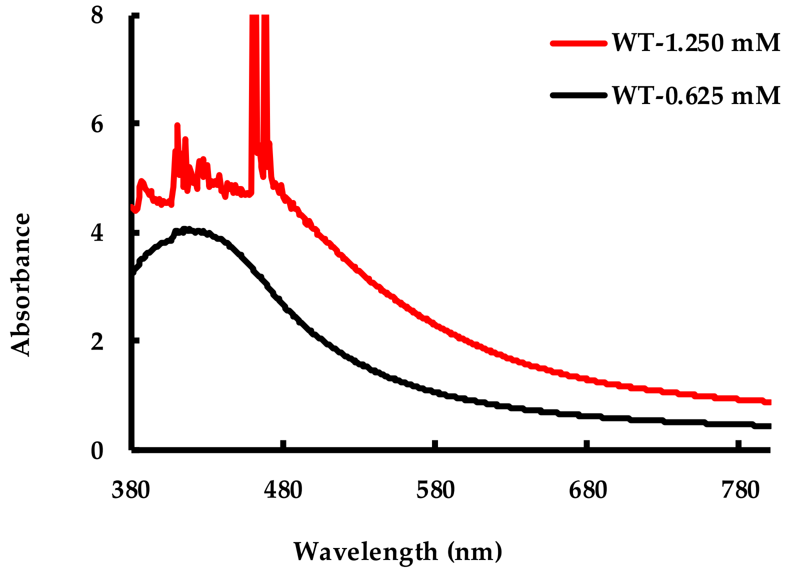

Figure S1. Saturated spectrophotometer reading by crude samples of WT-1.250 mM and WT-0.625 mM.

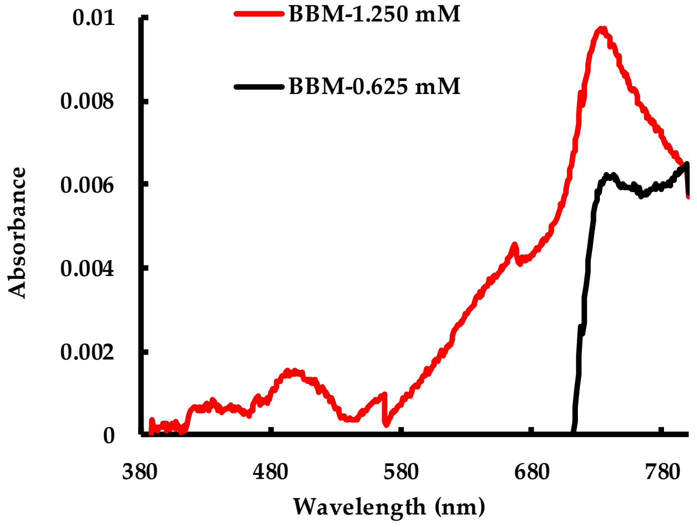

Figure S2. Spectrophotometric measurements of BBM + 1.250 mM and BBM + 0.625 mM.

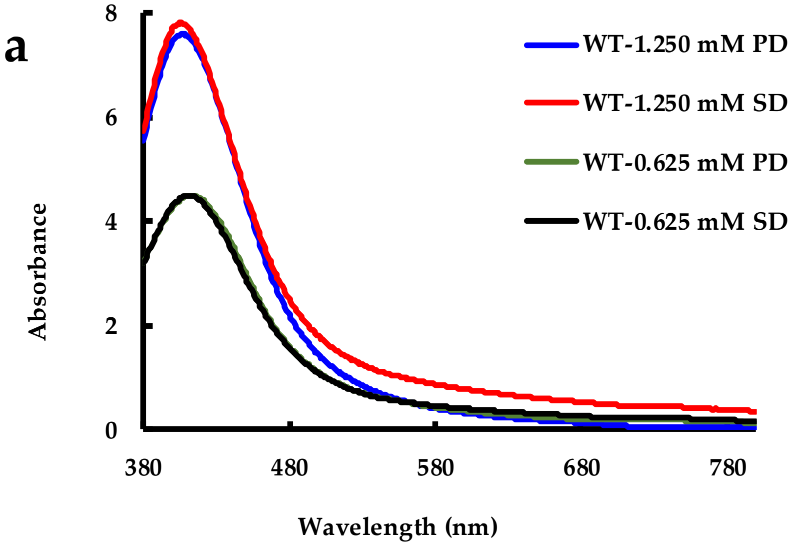

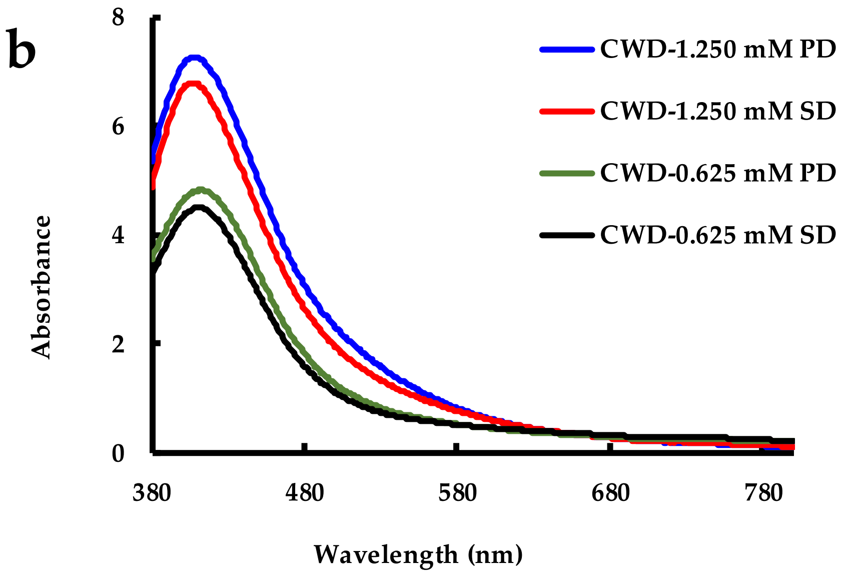

Figure S3. Spectrophotometric measurements of AgNPs, (**a**) produced by the Wild Type (WT) strain; and (**b**) produced by the Cell Wall Deficient (CWD) strain, being exposed to NaCl solutions for 72 h.
